# Comparison of intraoperative versus preoperative ERCP with laparoscopic cholecystectomy for cholecystocholedocholithiasis: a 3-year study at Kepler University Hospital

**DOI:** 10.1007/s00464-024-11438-x

**Published:** 2024-12-16

**Authors:** Sandra Raab, Alexander Jagoditsch, Franz Kurz, Philipp Pimingstorfer, Wolfgang Schimetta, Rainer Schöfl, Peter Schrenk, Christoph Schwinghammer, Alexander Ziachehabi, Andreas Shamiyeh

**Affiliations:** 1 Department for General-, and Visceral Surgery, Kepler University Hospital, Krankenhausstraße 9, Linz, Austria; 2 Medical Faculty, Johannes Kepler University, Linz, Austria; 3 Department of Internal Medicine and Gastroenterology, Kepler University Hospital, Linz, Austria; 4 Department of Applied Systems Research & Statistics, Johannes Kepler University, Linz, Austria; 5 Endoscopy Department, Phyrn-Eisenwurzen Hospital, Steyr, Austria; 6 Department of Gastroenterology, Hepatology, Endocrinology, Ordensklinikum, Linz, Austria

**Keywords:** Intraoperative ERCP, Preoperative ERCP, Cholecystocholedocholithiasis, Timing cholecystectomy

## Abstract

**Background:**

Preoperative ERCP followed by cholecystectomy is a common treatment for cholecystocholedocholithiasis. However, intraoperative ERCP has been used more frequently over the last two decades, with few studies assessing various aspects of both methods. We evaluated and compared the management and outcomes of intraoperative ERCP and preoperative ERCP.

**Methods:**

This is a retrospective cohort study of a prospective registry. A total of 169 patients with cholecystocholedocholithiasis underwent either intraoperative ERCP or preoperative ERCP followed by cholecystectomy. Between January 2020 and June 2023 patients were further analysed for morbidity, surgical technique, ERCP and surgical indications, length of stay and success rate of ERCP.

**Results:**

103 patients (60.9%) underwent intraoperative ERCP and 66 patients (39.1%) an ERCP later followed by cholecystectomy. Patients with intraoperative ERCP tended to have a lower rate of post-ERCP pancreatitis (3.9% vs. 6.1%; *P* = 0.537), fewer ERCP complications (3.9% vs. 10.6%; *P* = 0.116), a reduced hospital stay (8 vs. 13.8 days; *P* < 0.001) and a lower cannulation failure rate of the common bile duct during ERCP (1.9% vs. 6.1%; *P* = 0.088).

**Conclusions:**

Our study identified several advantages of intraoperative simultaneous ERCP over upfront ERCP, suggesting that intraoperative ERCP may be a viable and safe option for the comprehensive management of cholecystocholedocholithiasis.

Gallstones are common, affecting 10–15% in the Western population [1]. Common bile duct stones (CBDS) are detected in 4.6–15% (Europe) [2, 3] but may be as high as 20.9% in South America [4]. CBDS are usually found either via ultrasound, endosonography or magnetic resonance cholangiopancreatography (MRCP) [5]. Endoscopic retrograde cholangiopancreatography (ERCP) is used for the removal of stones from the common bile duct (CBD) since even asymptomatic gallstones in the CBD are associated with complications (pancreatitis, cholangitis, obstruction) of up to 25.3% within 4 years [2]. Historically, the standard approach for treating CBDS has been a two-stage access with ERCP for stone removal (preoperative ERCP) followed by cholecystectomy. A few older studies in the literature compared these two procedures with an advantage found of intraoperative ERCP [6–9]. The ESGE guidelines (European Society of Gastrointestinal Endoscopy) recommend considering an intraoperative ERCP for stone removal, but owing to the longer duration of surgery and shortness of equipment in the operating room (OR), ERCP is recommended prior to laparoscopic cholecystectomy. Laparoscopic cholecystectomy is then recommended within 2 weeks after ERCP because it is associated with a lower complication rate, such as bowel injuries, and a better defined Calots triangle anatomy [10, 11]. Recurrent biliary events after ERCP occur in 36.2% of patients with late cholecystectomy (6–8 weeks) compared to 2.1% when an early cholecystectomy (within 72 h) is performed [12]. In the SAGES (Society of American Gastrointestinal and Endoscopic Surgeons) guidelines intraoperative ERCP is not recommended but laparoscopic or open bile duct exploration is the standard of care [13]. According to the German S3 guidelines, intraoperative and preoperative ERCP are both regarded as treatment options. Due to logistic difficulties preoperative ERCP is recommended [14]. One disadvantage of intraoperative ERCP is the longer duration of surgery [15]. As those procedures are often emergent, scheduled surgeries could be postponed or cancelled. Preoperative ERCP, however, offers a more flexible approach.

This study aims to compare the outcomes of intraoperative ERCP with those of preoperative ERCP followed by cholecystectomy in the management of cholecystocholedocholithiasis, while outlining the respective advantages and disadvantages of each approach.

## Methods

### Study design

The study is a retrospective cohort study of a prospective registry (ClinicalTrials: NCT06130163). It was approved by the Johannes Kepler University (JKU) Linz ethics committee (1216/2023) and follows the STROBE guidelines [16].

### Patients

From January 2020 to June 2023, all patients admitted to hospital with cholecystocholedocholithiasis (CCL) who underwent intraoperative ERCP or preoperative ERCP followed by cholecystectomy were included. Age under eighteen years was the only exclusion criterion. The observation period of patients was from the first hospital admission, when CBDS were diagnosed, until the last discharge, when cholecystectomy was carried out (counting subsequent hospital stays due to complications of surgery/ERCP). ERCP was performed by experienced gastroenterologists or surgeons. CCL was diagnosed either with ultrasound (in suspicion of cholecystitis, CBD dilatation) or MRCP (in case of elevated bilirubin, gamma-GT, alkaline phosphatase). A computed tomography (CT) scan was performed in patients for whom ultrasound data were not available (initial presentation with acute abdomen, deceptive abdominal pain). To determine post-ERCP pancreatitis laboratory parameters were repeated the day after ERCP in every patient, pain was measured with a visual analogue scale, and ultrasound or CT scans were carried out if necessary.

### Outcome and variables

Primary outcomes of the study were post-ERCP pancreatitis (at least 2 of 3 criteria are fulfilled within 24 h after ERCP: amylase and lipase 3 times the upper limit of normal level, radiological signs of pancreatitis, abdominal pain) and surgical and ERCP complications due to surgery or ERCP (hemorrhage or cystic duct stump leakage).

Secondary outcome parameters were type of surgical method, conversion rate to open surgery, symptomatic and asymptomatic choledocholithiasis, common bile duct clearance, cannulation failure, length of hospital stay, lithotripsy and indications for surgery and ERCP. In the intraoperative (IO) group, hospital stay was counted from the day of admission until the day of discharge (one hospital stay), whereas length of stay in the preoperative ERCP group was calculated from the day of admission to discharge for ERCP and the second stay for cholecystectomy (two hospital stays).

### Technique of intraoperative ERCP

**Fig. 1:**
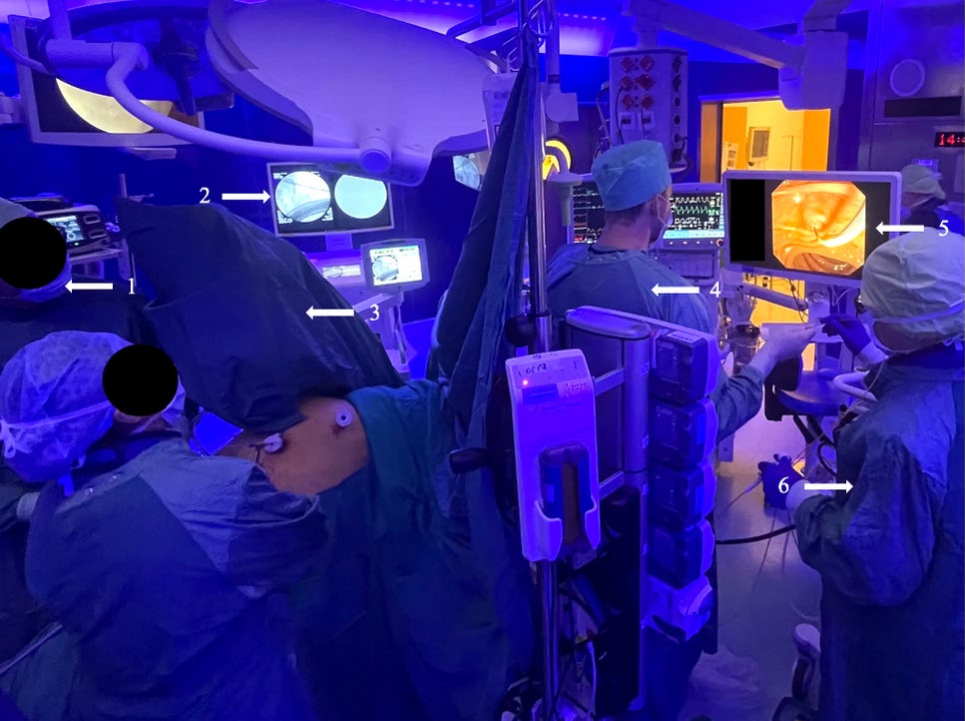
Operating room setup: 1 = surgeon, 2 = fluoro monitor, 3 = C-arm, 4 = endoscopist, 5 = endotower/guidewire passing through the papilla, 6 = assistant for the endoscopist

For an intraoperative ERCP, patients remain in the French position. The endoscopist stands on the left side of the patient next to the anesthesiologist, and all personnel in the operating theater adhere to the use of protective aprons. Before the endoscope is inserted, most of the dissection of the gallbladder is performed (Fig. 1). During intraoperative cholangiography (IOC) the surgeon inserts an antegrade guidewire into the cholangiography catheter, with the endoscopist performing the ERCP grasping the guidewire with a biopsy forceps upon passing the major duodenal papilla and pulling the wire through the duodenoscope channel (rendezvous cannulation). A sphincterotome is inserted along the guidewire in the CBD. After removing the endoscope the surgeon finishes the dissection of the gallbladder.

### Statistical analysis

Patient baseline characteristics were summarized using descriptive statistics. Categorical variables were compared using the two-sided Pearson *χ*^2^ test or Fisher exact test, as appropriate. Continuous variables were compared using the *t* test (normal distribution) or the Mann–Whitney *U* test (abnormal distribution). Logistic regression was used for univariate and multivariate analyses with 95% confidence intervals (CIs). Missing data were described as unknown and included in the analysis. All *P* values were two-sided and considered significant when < 0.05. The type I error was not adjusted for multiple testing. Statistical analyses were performed using the open-source R statistical software package, version 4.2.3 (The R Foundation for Statistical Computing, Vienna, Austria).

## Results

### Patients

Between January 2020 and June 2023, 169 patients were admitted to the Kepler University Hospital Linz and diagnosed with cholecystocholedocholithiasis. Among these patients, 103 patients underwent intraoperative ERCP (60.9%), and 66 patients had cholecystectomy with ERCP upfront (39.1%). Patient characteristics are shown in Table 1 and were comparable between the two groups (not significant). Table 2 shows the indications for ERCP as well as the type of surgical method performed.

**Table 1:** Patient characteristics of 169 patients treated for cholecystocholedocholithiasis *(BMI* body mass index in kg/m^2^, *ASA* American Society of Anesthesiologists physical status classification)

	Intraoperative ERCP	Preoperative ERCP
Total patients	103 (60.9%)	66 (39.1%)
Age, years, mean	62.6	63.8
*Sex*		
Male	48 (46.7%)	33 (50.0%)
Female	55 (53.3%)	33 (50.0%)
BMI, kg/m^2^, median	32.4	30.8
ASA, median	2	2
Preoperative serum bilirubin (mg/dL)	3.2	4.4
Preoperative MRCP, median	61 (59.2%)	43 (65.2%)
*Comorbidity*		
Cardiovascular	49 (47.6%)	26 (39.4%)
Diabetes	11 (10.7%)	8 (12.1%)
Respiratory	9 (8.7%)	10 (15.1%)

**Table 2:** Indications for surgery in patients with intraoperative ERCP or preoperative ERCP followed by cholecystectomy

	Intraoperative ERCP *n* (%)	Preoperative ERCP *n* (%)	*P* value
Cholangitis ± biliary pancreatitis	15 (14.6)	17 (25.8)	
Biliary pancreatitis or cholecystocholedocholithiasis	49 (47.5)	44 (66.7)	
Cholecystitis and choledocholithiasis	39 (37.9)	5 (7.6)	
Emergency surgery	57 (55.3)	18 (27.3)	< 0.001**
Asymptomatic CBDS	14 (13.6)	0 (0.0)	< 0.001**
Laparoscopic cholecystectomy	94 (91.3)	62 (93.9)	0.490 NS
Open cholecystectomy	4 (3.9)	1 (1.5)	0.218 NS
Conversion to open cholecystectomy	8 (7.8)	3 (4.6)	0.435 NS

*P<0.05; **P<0.01

### Outcome of cholecystectomy

Surgical approaches are listed in Table 2. An open approach was planned in patients with previous complicated surgeries or due to the presence of impacted stones in the CBD identified via preoperative imaging. Conversion to open surgery was necessary because of the severity of cholecystitis. Six patients in the IO group had postoperative intraabdominal hemorrhage from the liver bed (re-laparoscopy) and one patient had a cystic duct stump leakage undergoing Re-ERCP with stent placement. In the preoperative ERCP group surgical complications (postoperative hemorrhage) occurred in 2 patients necessitating re-laparoscopy (Table 3).

**Table 3:** Morbidity and differences across all patients with cholecystectomy and ERCP according to group allocation counting all hospital stays

	Intraoperative ERCP *n* (%)	Preoperative ERCP *n* (%)	*P* value
Surgical complications	7 (6.8)	2 (3.0)	0.503 NS
ERCP complications	4 (3.9)	7 (10.6)	0.116 NS
Post-ERCP pancreatitis	4 [of 102^a^] (3.9)	4 (6.1)	undefined NS
Clearance rate	96 (94.1)	59 (89.4)	0.180 NS
Cannulation failure	2 (1.9)	4 (6.1)	0.088 NS
Stent implantation	32 (31.1)	15 (22.8)	0.770 NS
Lithotripsy	3 (2.9)	1 (1.5)	0.879 NS
Length of stay	Average: 8, Median: 7	Average: 13.8, Median: 11	< 0.001**

*NS* Not significant

*P<0.05; **P<0.01

^a^One patient died during surgery with intraoperative ERCP due to sepsis and cholangitis already with intraoperative reanimation

### Outcome of ERCP

Intraoperative cholangiography is routinely conducted in our clinic and all patients obtain complete blood samples at admission (emergency and elective) to check for possible choledocholithiasis. We detected asymptomatic CBDS (no laboratory/radiological signs) in 14 patients in the IO group during cholangiography (Table 2). ERCP associated complications (except post-ERCP pancreatitis) in the preoperative ERCP group were bleeding of the major duodenal papilla (*n* = 4), post-ERCP cholangitis (*n* = 2) and perforation of the duodenum (*n* = 1) (Table 3). Postinterventional cholangitis was treated with Re-ERCP and placement of a biliary stent. Retroperitoneal perforation was also addressed endoscopically using a stent. ERCP related complication in the IO group was bleeding of the major duodenal papilla (*n* = 4). Bleeding of the papilla was treated with ERCP, no surgery was needed. Genererally, common bile duct clearance was confirmed with intraoperative cholangiography showing no filling defects. In 32 patients with intraoperative ERCP a stent was placed during the procedure (2 pancreatic stents, 30 common bile duct stents), which was later removed with gastroscopy. During preoperative ERCP 15 patients received a stent (6 pancreatic stents, 10 common bile duct stents). Post-ERCP pancreatitis occurred in 3.9% of patients in the intraoperative group and 6.1% in the preoperative ERCP group. ERCP was not successful in 2 patients with intraoperative ERCP due to impacted stones with unsuccessful insertion of the guidewire via the cystic duct and in one patient with status post Billroth II surgery with conversion to open choledochotomy. Four patients in the preoperative ERCP group had unsuccessful ERCP as probing the papilla was not possible, swelling of the papilla or undetectable papilla. Therefore, we either opted for an intraoperative ERCP with a guidewire, needle papillotomy or precutting of the papilla in a second session. Patients undergoing intraoperative ERCP had a significantly shorter hospital length of stay (*P* < 0.001) than patients with preoperative ERCP (both hospital stays combined) (Table 3).

## Discussion

**Fig. 2:**
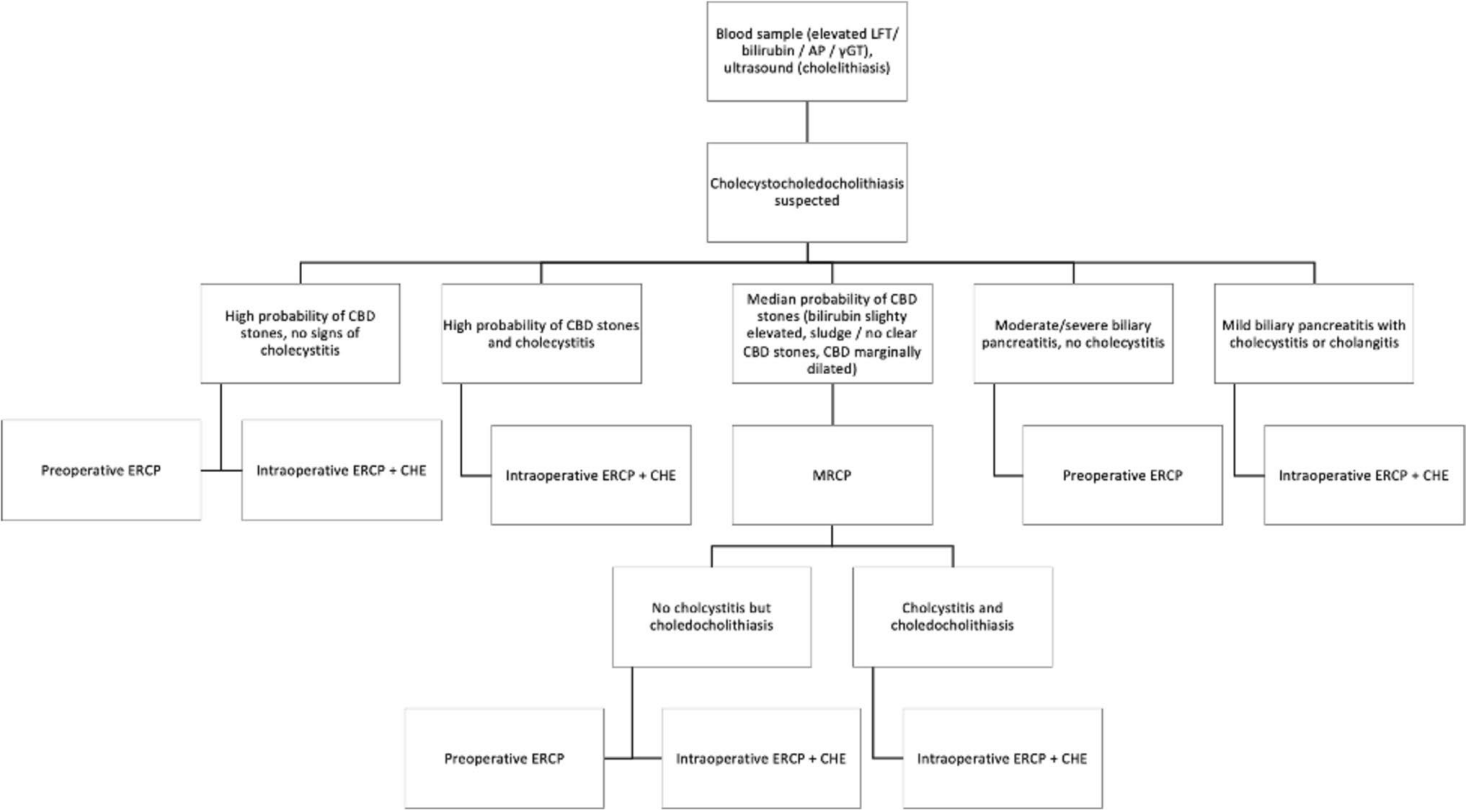
Algorithm for patients with suspected of having cholecystocholedocholithiasis (*LFT* liver function test, *AP* alkaline phosphatase, *γGT* gamma-glutamyl transpeptidase)

Cholecystocholedocholithiasis remains a challenging surgical problem, as advanced techniques in laparoscopy and endoscopy expand the indications for cholecystectomy with biliary duct clearance [17, 18]. Our results of the study revealed no significant differences between both groups with respect to complications. This may be due to the relatively low number of patients included. Randomization, however, may be difficult given the availability of an experienced surgeon/gastroenterologist to perform ERCP whenever needed. In our clinic, we started in 1990 conducting preoperative ERCP for cholecystocholedocholithiasis [19]. To exclude cholecystocholedocholithiasis, all patients with cholecystolithiasis get ultrasound and blood sampling including liver function tests and total bilirubin, gamma-glutamyl transpeptidase, alkaline phosphatase, amylase and lipase. If those two examinations reveal a greater probability of CBDS (dilated CBD, elevated bilirubin, elevated γGT/AP/LFT, ultrasonic proof of CBDS, signs of cholangitis), patients directly undergo either preoperative ERCP or intraoperative ERCP. Otherwise, MRCP or endosonography is recommended for further diagnosis, as shown in Fig. 2 based on the S3 guidelines [14]. In patients with mild biliary pancreatitis, cholecystectomy is performed as soon as possible, whereas during severe pancreatitis, ERCP is performed upfront and cholecystectomy after the remission of pancreatitis.

In our results we mentioned a high incidence of asymptomatic CBDS with 13.6% without any signs in the blood samples [3]. This could be due to patients having concomitant sludge in the common bile duct. These patients had no postoperative complications. However, a management algorithm for removing CBDS, especially when asymptomatic remains individualized. Möller et al. suggested removing all sizes of stones in the CBD with regard to complication rate. For stones smaller than 4 mm, complications depend on the technique used to remove the stones (higher for postoperative ERCP, open choledochotomy) [2]. According to Collins et al. 26% of CBDS during intraoperative cholangiography are false positive, and an additional 26% pass spontaneously [3]. Anyway, intraoperative cholangiography is necessary when MRCP or endosonography is not available. Another reason for conducting intraoperative cholangiography routinely is to gain experience in interpreting fluoroscopy in case of altered anatomical situations. In our clinic, both surgeons and gastroenterologists perform ERCP, and an endoscopist is always available during the week and also on weekends in case of emergency. Nevertheless, the choice of treatment depends on the level of experience and availability at each hospital [20].

Another advantage of intraoperative ERCP (Table 4) is the reduced hospital stay. Owing to its lower overall morbidity and shorter length of stay Liao et al. endorsed intraoperative ERCP [9]. The literature proposes lower costs and a single-stage treatment [8]. Average length of stay in our study was 8 days in the intraoperative group and 13.8 days in the preoperative ERCP group and was a significant difference. Patients undergoing elective cholecystectomy are typically admitted to the hospital the day before the procedure and remain at least two days postoperatively. The length of stay after emergency surgery depends on the severity of the inflammation and may also be extended due to comorbidities. Patients are frequently on oral anticoagulation delaying interventions. In case of pancreatitis, treatment typically involves fluid resuscitation followed by the resumption of an oral diet [21].

**Table 4:** Summary of the advantages and disadvantages of an intraoperative ERCP

Advantages	Disadvantages
Short hospital length of stay	Operating theater capacity
Lower costs	More difficult surgery
Single-stage treatment	Poor equipment
Easier cannulation of papilla	
Higher cannulation success	
Option for choledochotomy	
Salvage when preoperative ERCP fails	
Access to gastric remnant	

A well-known advantage of intraoperative ERCP is the lower rate of post-ERCP pancreatitis [15, 22, 23]. Using antegrade guidewires, three potential risk factors for post-ERCP pancreatitis can be reduced: retrograde pancreatic cannulation, injection of contrast media into the pancreatic duct and prolonged manipulation of the papilla, which may all be responsible for the lower rate of pancreatitis [24]. As previously described in the literature, we also reported a tendency for more successful cannulation (98.1% vs. 93.9%) [25]. If intraoperative ERCP is unsuccessful, laparoscopic/open choledochotomy or postoperative ERCP are possible options [26]. For patients with advanced cholecystitis, laparoscopic cholecystectomy may be converted to open cholecystectomy. Therefore, it is not possible to compare conversion rate in both groups.

Additionally, the prevalence of bariatric surgery is on the rise, with gastric bypass procedures making it challenging to perform an ERCP for CBDS due to altered anatomy. In this situation, intraoperative ERCP is the preferred choice, especially when the gallbladder is in place [27].

A disadvantage of intraoperative ERCP is a longer operation time and reduced operating theater capacity [15].

We would recommend intraoperative ERCP in patients with incidentally diagnosed CBDS by intraoperative cholangiography and cholecystitis with cholangitis whereas moderate or severe biliary pancreatitis may represent a contraindication.

A limitation of our study is the retrospective analysis of patients in an experienced single-center. However, due to comorbidities (e.g., oral anticoagulation) of patients who present with sepsis and cholecystocholedocholithiasis it may be impossible to establish a randomized trial. The reasons why our study did not reach statistical significance may be the sample sizes and distinct cohort sizes. We did not analyse the costs of different treatment approaches. It is uncertain whether length of stay and procedural costs influence general expenses, which needs further evaluation.

In conclusion, this study provides several advantages of an intraoperative ERCP in managing cholecystocholedocholithiasis. Our data further confirm that intraoperative ERCP is a safe procedure, presenting no additional surgical or endoscopic risks compared with preoperative ERCP. However, we believe that intraoperative ERCP can be a viable option once logistic obstacles, such as interdisciplinary collaboration, are overcome.

## Abbreviations

CCL: Cholecystocholedocholithiasis
ERCP: Endoscopic retrograde cholangiopancreatography
CBD: Common bile duct
CBDS: Common bile duct stones
MRCP: Magnetic resonance cholangiopancreatography
IO: Intraoperative
CT: Computed tomography
