# Correction: Comparison of intraoperative versus preoperative ERCP with laparoscopic cholecystectomy for cholecystocholedocholithiasis: a 3-year study at Kepler University Hospital

**DOI:** 10.1007/s00464-024-11507-1

**Published:** 2025-01-09

**Authors:** Sandra Raab, Alexander Jagoditsch, Franz Kurz, Philipp Pimingstorfer, Wolfgang Schimetta, Rainer Schöfl, Peter Schrenk, Christoph Schwinghammer, Alexander Ziachehabi, Andreas Shamiyeh

**Affiliations:** 1 Department for General-, and Visceral Surgery, Kepler University Hospital, Krankenhausstraße 9, 4020 Linz, Austria; 2 Medical Faculty, Johannes Kepler University, Linz, Austria; 3 Department of Internal Medicine and Gastroenterology, Kepler University Hospital, Linz, Austria; 4 Department of Applied Systems Research & Statistics, Johannes Kepler University, Linz, Austria; 5 Endoscopy Department, Phyrn-Eisenwurzen Hospital, Steyr, Austria; 6 Department of Gastroenterology, Hepatology, Endocrinology, Ordensklinikum, Linz, Austria

**Correction to: Surgical Endoscopy (2024)** 10.1007/s00464-024-11438-x

The original online version of this article was revised to correct the order of coauthors.

The original article has been corrected.

